# Retraction: The influence of evidence-based nutritional support plans on the nutritional status and adverse effects of radiotherapy in individuals with nasopharyngeal carcinoma

**DOI:** 10.3389/fnut.2026.1932744

**Published:** 2026-07-13

**Authors:** Frontiers Editorial Office

The Journal retracts the 15 April 2025 article cited above.

Following publication, concerns were raised regarding the scientific validity of the article. An investigation was conducted in accordance with Frontiers' policies. The authors failed to provide a satisfactory explanation and as a result, the conclusions of the article have been deemed unreliable, and the article has been retracted.

This retraction was approved by the Chief Editors of Frontiers in Nutrition and the Chief Executive Editor of Frontiers. The authors agreed to this retraction. Frontiers would like to thank the users on PubPeer for bringing the published article to our attention.

